# Condition Classification of Water-Filled Underground Siphon Using Acoustic Sensors

**DOI:** 10.3390/s20010186

**Published:** 2019-12-28

**Authors:** Xuefeng Zhu, Guoyong Huang, Zao Feng, Jiande Wu

**Affiliations:** 1 Faculty of Information Engineering and Automation, Kunming University of Science and Technology, Kunming 650000, China; zhuxuefeng@stu.kust.edu.cn (X.Z.); fengzao0923@hotmail.com (Z.F.); wjiande@kust.edu.cn (J.W.); 2 Yunnan Key Laboratory of Artificial Intelligence, Kunming University of Science and Technology, Kunming 650500, China

**Keywords:** water-filled underground siphons, acoustic sensors, condition classification, VMD, density of sound energy

## Abstract

Siphons have been widely used in water supply systems and sewage networks. However, it is difficult to implement non-destructive testing due to structural complexity and limited accessibility. In this paper, a novel condition classification method for water-filled underground siphons is proposed, which uses the acoustic signals received from acoustic sensors installed in the siphon. The proposed method has the advantages of simpler operation, lower cost, and higher detection efficiency. The acoustic wave forms in the siphons reflect on the system characteristics. Seven typical conditions of a water-filled underground siphon were investigated, and a series of experiments were conducted. Acoustic signals were recorded and transformed into acoustic pressure responses for further analysis. The variational mode decomposition (VMD) and the acoustic energy flow density were used for signal processing and feature extraction. The acoustic energy flux density eigenvectors were input to three different classifiers to classify the siphon conditions. The results demonstrate that the proposed acoustic-based approach can effectively classify the blockage and damage conditions of siphons, and the recognition accuracy of the proposed method is higher than 94.4%. Therefore, this research has value for engineering applications.

## 1. Introduction

A complex infrastructure network above and under the ground can determine the economic prosperity of modern cities. Water supply systems (WSS) and sewage are the core of urban underground networks [1,2]. Siphons can transport sewage and water run-off under deep obstacles without a continuous gradient at the intersection of pipelines, such as railway lines, high-rise buildings, and bridges. Siphons are often vulnerable to damage due to their long service life, corrosion of aging, structural complexity, and external load impact. There are two types of damage during the service life of siphons, one is the structural defects caused by the quality of siphons or external influences, including deformation, leaking, corrosion, and foreign material [3]. The other is caused by changes in the physical and chemical environments in siphons, including functional defects resulting from the intrusion of external substances, such as scale, deposits, obstacles, and scum [4]. Therefore, it is especially necessary to identify siphon conditions to ensure the safety and reliability of buried siphons. The unqualified environment in siphons decreases the efficiency of manual operation and increases the safety risk, and it is of considerable significance to investigate the methods of assessing urban buried siphons in order to minimize the use of manual detection.

Recently, a series of methods have been developed for blockage or leakage detection in pipes, such as piezoelectric ultrasonic sensors technology [5], eddy current probes [6], closed-circuit television (CCTV) [7], sewer scanner evaluation technology (SSET) [8], and ground-penetrating radar (GPR) [9,10]. Piezoelectric ultrasonic sensors can detect minimal defects, and they have the advantages of being portable, non-polluting, and highly accurate for pipeline damage detection. However, piezoelectric ultrasonic testing is relatively difficult, tedious, and expensive, and its use is limited by the operating frequency. Eddy current probe detection has several limitations, and it can only be used to detect the surface or near-surface defects in magnetic or non-magnetic conductive siphons. In CCTV and SSET, the camera is installed on a mobile robot, which passes through the siphon to obtain the video data. The images can be used to manually or automatically evaluate the health or structure of siphons. Nevertheless, the detection is expensive and limited to relatively small portions of the sewer system.

In contrast, acoustic detection is a non-destructive testing method with the advantages of simple implementation, low cost and high detection efficiency. The detection results do not depend on the subjectivity of the inspectors. Acoustic detection is suitable for inspecting pipes with different shapes (straight, curved, and siphon) and fluids of different densities (low, medium, and high). The changes in acoustic wave forms are related to the system characteristics (such as blockage and leakage) [11,12]. According to recent study results, the detection methods can detect sewer blockages and leakages based on acoustic signals. Consequently, the US Environmental Protection Agency’s Office of Research and Development developed the portable and rapidly deployable sewer line rapid assessment tools SL-RAT^TM^ [13] and Sewer-Batt^TM^ system [14] and conducted a series of experiments. The SL-RAT^TM^ system predicts the blockage degree in pipes by transmitting a multi-tone acoustic signal to the pipes and measuring the attenuation of the transmission signal. The Sewer-Batt^TM^ system determines the acoustic response according to the intensity of the reflected signal, and works well for detecting pipe blockages with or without side gates. However, a CCTV system is required for further detection due to its limitations with respect to multiple defect recognition.

In this paper, we propose a detection method based on acoustic sensors. Compared with ultrasonic signals, low-frequency acoustic signals with less attenuation are actively transmitted into siphons.

1. The active acoustic detection method was adopted. A sinusoidal sweep signal was used as the excitation signal, and the sound pressure data were collected at the receiving end to measure the acoustic impulse response of the pipe.
2. VMD was employed to decompose acoustic signal components and obtain the band-limited intrinsic mode function (BIMF). Mutual information was introduced to quantitatively characterize the BIMF components. The components containing the most information about the siphon conditions were chosen, so that the effective feature information can be kept. According to the mutual information screening coefficient, the effective BIMF components were selected to reconstruct the signal. The root means square error of the reconstructed signal and the original signal was calculated. Moreover, the optimal result of component number *k* is determined using the minimum root mean square error.
3. The BIMF component was conducted with sound pressure level (SPL) transformation, and the acoustic energy flow density value of the filtered BIMF component was extracted as the classification feature. Finally, the eigenvalues were input to three classifiers, namely, random forest (RF), k-nearest neighbor (KNN), and support vector machine (SVM). The operation accuracy of the three classifiers for identifying the siphons conditions is higher than 94.4%, which verifies the effectiveness of identifying siphon conditions based on acoustic features.

## 2. Related Theories

**Figure 1:**
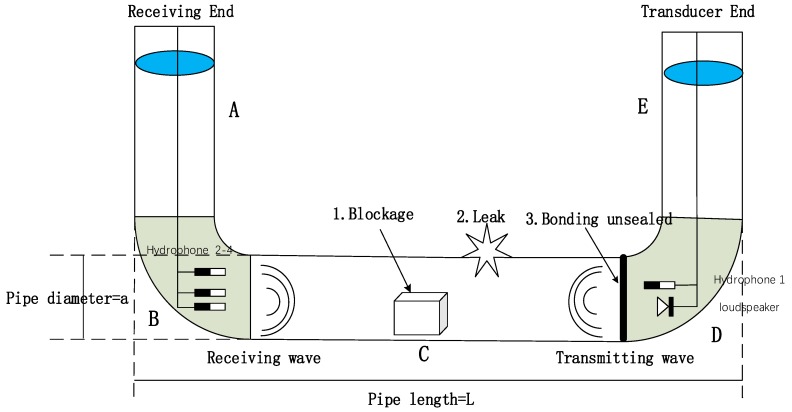
Acoustic wave detection in the siphon.

A siphon is composed of two elbows (B, D) and three straight pipes (A, C, and E), as shown in Figure 1. The material of siphon pipes determines the hydraulic characteristics of the composite elbow. Because of the density difference between solid particles and water in a siphon, the centrifugal force also acts on the siphon, the pressure on the outer wall rises, and the pressure on the inner wall decreases. Due to the lower pressure on the outer wall correspondingly and the higher pressure on the inner wall, an eddy current zone is formed near the inner wall, causing three-dimensional diffusion. The particles enter elbow B and come into contact with the pipe wall outside the elbow. Subsequently, the particles are bounced into water, collide with the pipe wall a second time, and flow with the water in the elbow. However, a particle-free area is formed on the inner side of elbow B, and a large number of particles are accumulated on the outer side of the elbow, thereby reducing the effective area of the mainstream, causing accumulation of solid particle waste. Moreover, blockages are formed in section C of the siphon. The undulations continue and the residual water remains in front of the blockage, which affects the safe operation of the drainage pipe and increases the hidden danger of the siphon.

The joint between the elbow and the straight of the siphon is connected by bonding. When there is water in the siphon, a stress joint will be generated near the bonding seam of the bend. The stress joint is near the bend, and the corrosion in the pipe quickly leads to stress corrosion leaking at the bonding location. During the operation of the siphon, the bonding site is continuously impacted by granular impurities. In the case of low velocity, the particles have less impact on the pipe wall, and the wear on the pipe wall is small. As the increase of the velocity, the radial velocity gradient of the particles in the pipe is higher, and the collision between the particles and the pipe wall is intensified, which enhances the wear degree of the pipe wall. As a result, an unsealed joint is likely to occur.

According to the pipe acoustics theory, as the siphon has a linear-elastic stress–strain relationship and a constant boundary stat, the incident sound wave propagates in the siphon due to vibration and the velocity is the same at all locations [15]. The changes in acoustic impedance caused by the abrupt change in the cross-sectional area will result in interface reflection, interference, and diffraction, thereby increasing the propagation path of the sound wave. More time will be taken for sound transmission, and the energy dissipation (amplitude reduction) is increased [16,17]. Research on siphon damage has shown that the change in stiffness is closely related to the type and degree of damage. Damage degree can be determined by monitoring the change in stiffness. However, it is difficult to directly measure the stiffness, which is generally reflected by the dynamic response characteristics of the structure. Consequently, the acoustic signal transmitted in the pipe can reflect the stiffness change and determine the damage status [15].

The radius of the pipe is *a*, the length is *L*, and the plane wave *x* is incident along the direction of the plane. The angle between the wave vector *x* and the axis is *θ_i_*. We assume that both ends of the cylindrical pipe with a finite length meet the boundary conditions, and the influence of sound scattering of the cylindrical pipe is ignored. There is a blockage in the pipe, or the pipe wall is damaged. The sound field of the cylindrical pipe is:

(1) $$P_{e}=p_{i}+p_{rig}+p_{res}$$

where $p_{i}$ is the incident sound field, $p_{i}=e^{i\left(k_{z}z+k_{x}x-wt\right)}$, $k=w/c_{0}$ is the wave number of the sound wave in the water in the pipe. $p_{rig}$ is the scattering sound field caused by a rigid structure, and $p_{res}$ is the scattering sound field caused by a flexible fabric. In the siphon, the pipe wall has a rigid structure, and the plug has an elastic structure.

In a normal condition, the material of the pipe is even and dense. The sound wave is refracted and scattered in the pipe, and it propagates from the transmitting end to the receiving end in the shortest time. In this case, the attenuation of sound wave energy is the least. In the case of a blockage in the siphon, the sound wave is refracted and scattered, and the blockage is diffracted. Since the blockage is generally filled with air or water vapor, the characteristic impedance of the blockage and the pipe wall, air, or water vapor are significantly different. The low-frequency sound wave is diffused and propagated bypassing the blockage. The propagation path is longer, the sound becomes more extensive, and the waveform changes. The sound wave is received by the receiving end after passing through the blockage. When a blockage exists in the pipe, sound pressure and reverberation scattering are generated, thereby increasing the amplitude of the sound wave. The backscattered sound field caused by the blockage is as below [18]:

(2) $$p_{res}=\frac{e^{ikd_{0}}}{d_{0}}\frac{2i\rho_{0}\omega L}{\pi^{2}k_{x}\alpha }\overset{\infty }{\underset{n=0}{\sum }}\overset{\infty }{\underset{p=1}{\sum }}\varepsilon_{n}{\left(\frac{k\;sin\theta_{i}}{\alpha_{p}}\right)}^{n}\frac{F^{2}\left(k\;cos\theta_{i}L,k_{p}L\right)}{\alpha_{P}H_{n}^{1}\left(\alpha_{p}\alpha \right)H_{n}^{1}\left(k_{x}\alpha \right)Z_{np}}{\left(-1\right)}^{n}$$

The scattered sound field created by the tube wall is [18]:

(3) $$p_{rig}=\frac{e^{ikd_{0}}}{d_{0}}\frac{i}{\pi k}\frac{sin\left[2kL\;cos\theta_{i}\right]}{cos\theta_{i}}\overset{\infty }{\underset{n=0}{\sum }}\varepsilon_{n}\left[\frac{J_{n}^{\prime }\left(k_{x}\alpha \right)}{H_{n}^{1}\left(k_{x}\alpha \right)}\right]{\left(-1\right)}^{n}$$

During the propagation of an acoustic wave in the pipe, as the propagation distance increases, the energy is attenuated because the sound waves are scattered and absorbed. If there is a leak in the siphon, the acoustic energy dissipation increases. When congestion occurs in the siphon, the energy is absorbed and the amplitude of the wave decreases, resulting in the fluctuation of siphon energy. As blockage or leakage occurs in the siphon, the sound energy changes, which is reflected as the changes in the sound energy flow density per unit time through the single-plane area perpendicular to the direction of energy propagation.

In an ideal medium, the sound energy per unit volume is the sound energy density, and its expression is as follows:

(4) $$E=\frac{1}{2}\rho_{0}V^{2}+\frac{1}{2}\frac{P^{2}}{\rho_{0}c^{2}}$$

where c is the propagation velocity of the sound wave in the medium, $\rho_{0}$ is the density of the medium, P is the sound pressure, and V is the particle vibration velocity.

In the present study, the acoustic energy flow density is calculated by using the root-mean-square method. The sound wave is a circularly symmetric beam in the pipe. The acoustic energy flow density is defined as follows:

(5) $$\omega =\frac{1}{Z}\int_{T_{P}}p^{2}\left(t\right)dt$$

where Z is the characteristic acoustic impedance of water. $Z=1.5\times 10^{6}\;kg\cdot m^{-2}\cdot s^{-1}$ is the instantaneous sound pressure at the time, and p(t) is the positive time integral limit. $T_{p}$ is the time between the first time when the sound pressure of the emitted sound wave exceeds 10% of the maximum value and the first time when it falls to 10% of the maximum value.

U(t) is the voltage generated by the hydrophone at time t, $U_{rsm}$ is the root mean square of the voltage in the time period $T_{p}$, p(t)=U(t)/M, and *M* is the sensitivity of the hydrophone. Equation (6) is obtained after discretization:

(6) $$\omega =\frac{U_{rsm}{}^{2}\cdot T_{P}}{Z\cdot M^{2}}$$

## 3. Experiments

**Figure 2:**
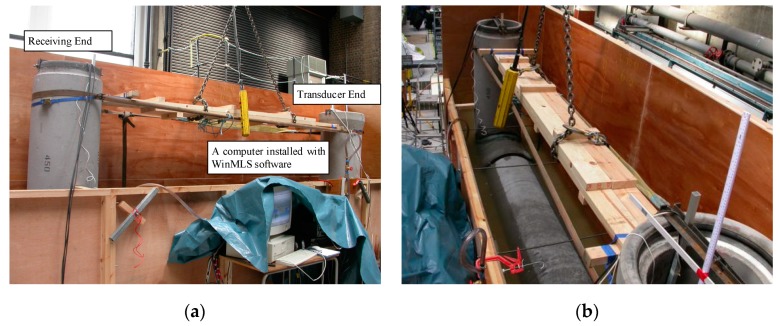
Photograph of the Siphon Experimental setup in the Hydraulics Laboratory at the University of Bradford.

A 450-mm diameter concrete siphon constructed in the Hydraulics Laboratory at the University of Bradford was used to carry out the experiments [16]. The acoustic signal acquisition system consists of hydrophones, a loudspeaker, a power amplifier, and a computer running WinMLS software. As shown in Figure 2a, a siphon with the length of 420 mm, height of 200 mm high, and diameter of 450 mm was installed on a layer of fine sand 500-mm thick in an open-top box made of 12 mm plywood. In all experiments, the siphon was fully surrounded by dry sand and was filled with water to simulate real live sewer conditions, the water level of 900 mm below the top rims of the vertical parts (Figure 2b).

Four hydrophones were used for acoustic detection. As shown in Figure 1, hydrophone 1 was driven by a 100–6000 Hz sinusoidal sweep, which was generated by a computer running WinMLS software to control the sound card. Unwanted low-frequency machinery noise was removed from the signals received from the hydrophones H2–H4 by using three 8-channel high-pass hydrophone filters. A B&K Type 2610 amplifier and a dual variable Kemo VBF 10M filter were used to process and filter the signal received on the reference hydrophone in the 100–4000 Hz range. In addition, the underwater speaker was driven by a B&K Type 2708 power amplifier. The quality of the signal generated by the underwater speaker was subjectively controlled by a Rotel Type RA-9708 X Stereo amplifier and headphones.

The operator used a computer running WinMLS software to control the sound card to generate 10 s of a sine sweep signal in the frequency range of 50–6000 Hz. The acoustic signals propagating in the siphon were received by hydrophones (type SQ31) installed on the left side of the siphon. A K50WP speaker was installed on the right side of the siphon to test the effectiveness of the acoustic signal generated by Hydrophone 1. The sampling frequency was 22,050 Hz. WinMLS software was used to de-convolve the original acoustic signals received by hydrophones H1–H3 to obtain the acoustic pressure response. The original acoustic signals received on hydrophones H1–H3 were de-convolved with WinMLS software to obtain the acoustic pressure response.

**Figure 3:**
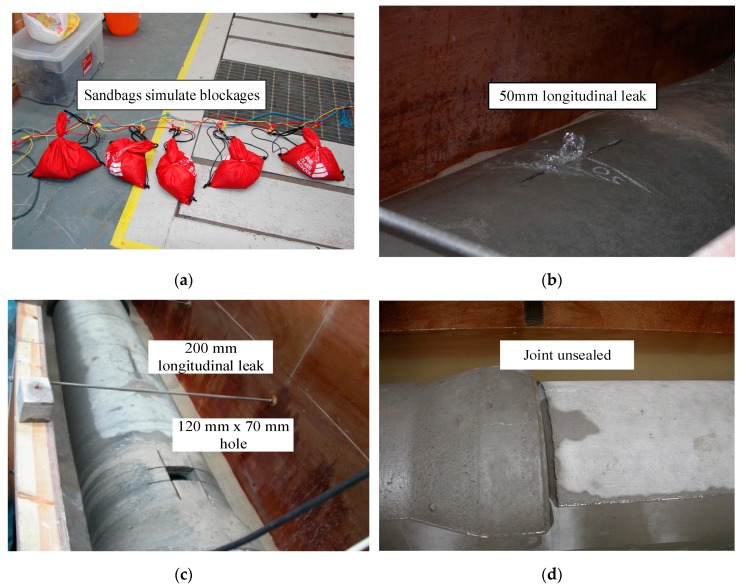
Siphon conditions simulated in the laboratory; (**a**) sandbags, (**b**) 50 mm longitudinal leak, (**c**) 200 mm longitudinal leak and 120 mm × 70 mm hole, (**d**) joint unsealed.

This experiment was carried out in order to investigate the influence of blockage, leakage and joint unsealed in the siphon. Several possible conditions were designed, as shown in Figure 3a. Ten 5 kg acoustically transparent bags were prepared and filled with fine sand to simulate the blockage in the siphon. The maximum cross-sectional area of a sandbag corresponded to approximately 20% of the siphon’s cross-section. Several bags were tied to a 9-m long rope with a distance of 300 mm. The number of bags deposited in the siphon varied from 1 to 10.

**Figure 4:**
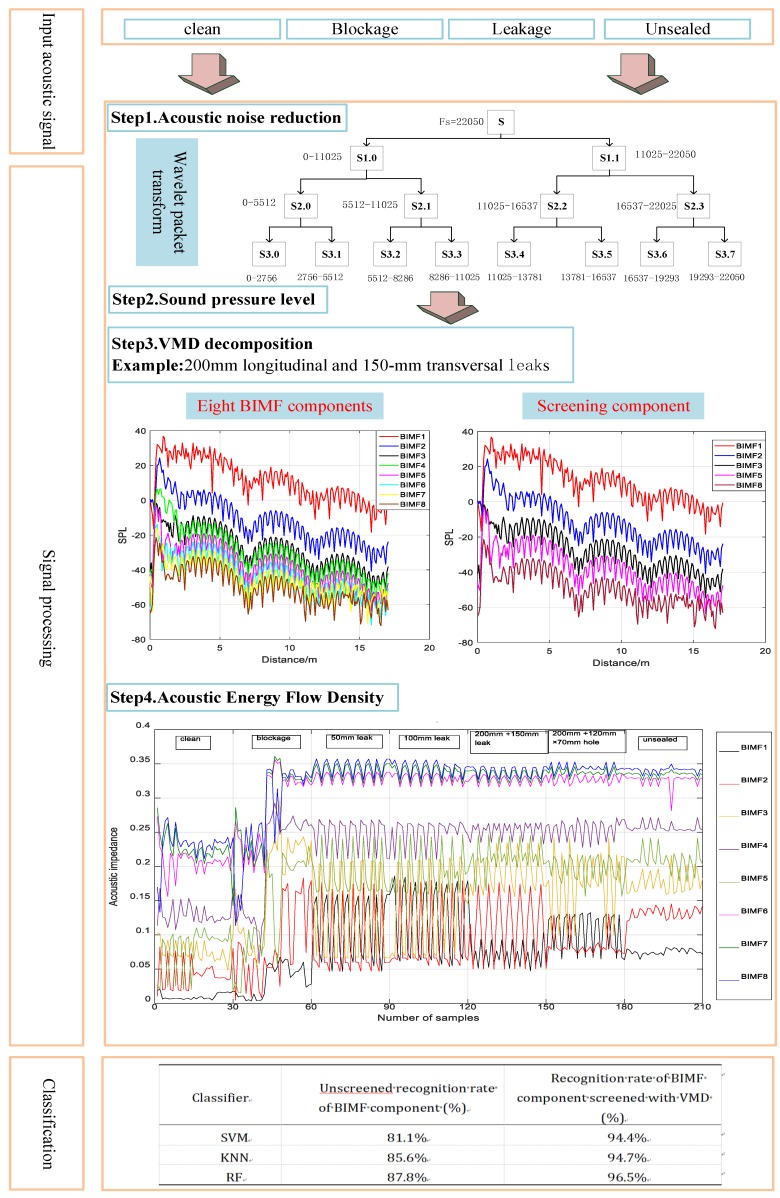
The flow diagram of our proposed approach.

Before additional measurements were made, the acoustic sensors were removed and re-installed when the bags were deposited or removed from the siphon. Subsequently, artificial cuts were made at top of the horizontal section of the siphon to simulate leaks. The siphon remained exposed and was surrounded by water up to the level matching the reference level inside the siphon after the damages were done. Measurements were taken in seven conditions: (1) clean siphon; (2) siphon with a controlled amount of blockage (Figure 3a); (3) siphon with a 50 mm longitudinal leak (Figure 3b); (4) siphon with a 100 mm longitudinal leak; (5) siphon with 200 mm longitudinal and 150-mm transversal leaks; (6) siphon with a 200mm longitudinal leak and a 120 mm × 70 mm hole (Figure 3c); and (7) joint unsealed (Figure 3d).

## 4. Proposed Feature Extraction Method

There are several intuitive methods to identify the siphon conditions from the collected sound pressure signals, i.e., (1) waveform distortion, (2) resonance peak in the frequency domain, and (3) waveform fluctuation characteristics. Due to the differences in siphon conditions, the sound pressure signal exhibits different fluctuations in amplitude, phase, intensity, extension width, azimuth, phase, etc. However, during the production, processing, and transmission of acoustic signals, the signal is affected by different types of noise. Therefore, to obtain the characteristic information of blockage or leakage in siphons from the acoustic signal, it is necessary to decompose a complex acoustic signal into several regular simple modes, which can be easily analyzed in the time and frequency domains. The purpose of feature extraction is to further classify the conditions.

Pattern recognition can extract feature information from a limited inspection record, allowing for the prediction of a siphon condition that has not been inspected [17]. Regardless of the method used for signal processing, it is necessary to accurately identify the frequency band containing siphon conditions in the acoustic signal. To date, feature extraction in signal processing has been conducted in the time domain, frequency domain, and time-frequency domain. Time-frequency domain methods which can locate both frequency and time have been extensively used, such as local mean decomposition (LMD), integrated empirical mode decomposition (EEMD), empirical mode decomposition (EMD), empirical wavelet transform (EWT), wavelet packet transform (WPT), wavelet transform (WT), and short-time Fourier transform (STFT) and [19,20]. Dragomiretskiy et al. proposed an adaptive decomposition method called variational mode decomposition (VMD) in 2014 [21]. This method has a solid foundation of mathematical theory. Compared with adaptive methods such as EMD and LMD, the VMD has fast convergence and good noise robustness. A Lagrange multiplication operator, a quadratic penalty factor, and a constrained variational model expression are used in the decomposition method. By transforming the binding variational problem into a non-binding variational problem, the mathematical significance is obtained, and the modal aliasing of the band-limited intrinsic mode function is minimized. However, the decomposition result of the VMD is affected by the number of modes *k* and the quadratic penalty factors *α*.

VMD and acoustic energy flow density are applied to assess and classify water-filled underground siphon conditions. The steps are as follows:

### 4.1. Step 1: Acoustic Noise Reduction

Due to the non-linearity and non-stationarity of acoustic signals, it is difficult to identify useful signals and denoise the data using a traditional Fourier transform. As an improvement of the Fourier transform, the WPT (wavelet packet transform) provides a more precise signal analysis method, which is more suitable for time-frequency analysis. Three-layer wavelet packet decomposition was used to decompose the acoustic signal. The appropriate wavelet packet frequency band was selected to reconstruct the signal by referring to the background noise signals of different siphon conditions, thus removing noise from acoustic signals.

The wavelet packet transform process is shown in Step 1 in Figure 4:

1. The layer number of decomposing the original signal is decomposed using an appropriate wavelet basis function;
2. An appropriate threshold value is selected to process the wavelet coefficients of each layer, and the processed wavelet coefficients are determined to obtain the denoised signal.

In this study, the sampling frequency of the acoustic pressure response is 22050 Hz, and the decomposition scale of the wavelet packet is 3. The wavelet packet is decomposed into eight signals in different frequency bands, including [0–2756], [2756–5512], [5512–8268], [8268–11024], [11024–13780], [13780–16536], [16536–19292], and [19292–22050]. The process of wavelet packet transform is shown in Step 1 of Figure 4, where S is the original signal, and $S_{i,j}$ is the wavelet steamed stuffed bun space (i is the number of decomposed layers, j is the subspace sequence of the first layer). The high-frequency part can be further decomposed through wavelet packet decomposition, and the original signal is decomposed into $2^{j}$ wavelet steamed stuffed bun space (j is the wavelet packet decomposition scale), forming a complete binary tree structure.

### 4.2. Step 2: Sound Pressure Level

At high amplitude component, the SPL [22] increases the lower amplitude component of acoustic signals, so that the characteristic signal hidden in the low-amplitude noise can be observed. In the present study, this method was used to amplify the content of the acoustic signal, thereby increasing the differences between different blockage degrees and facilitating the extraction of different siphon conditions in the subsequent decomposition. The calculation equation for the SPL is as follows:

(7) $$L_{p}=20\;lg\frac{P_{e}}{P_{0}}$$

where $p_{e}$ is the practical value of the sound pressure of the original sound wave signal, and $p_{0}$ is the practical value of the reference sound pressure. In this paper, $p_{0}=1\times 10^{-5}\;Pa$. The SPL transform process is shown in Step 2 in Figure 4.

### 4.3. Step3: VMD Decomposition

VMD has been used to extract features from non-stationary signals. The number of modes (*k*) and the quadratic penalty factors (α) should be selected empirically. One objective of this study is to select *k* according to the mutual information and the root mean square error. Mutual information, which is defined as the difference between two random variables in uncertainty, is used to measure the statistical correlation of two random variables. The advantage of mutual information is that it describes both the linear correlation and nonlinear correlation between two variables.

The steps of selecting VMD parameters are as follows:

1. The preset scale K1 is initially determined by observing the original signal of EMD (Empirical Mode Decomposition) decomposition and obtaining the maximum value for the first time. In this paper, the maximum value of K is 15.
2. The scope and step length of *k* and α are determined. In this study, the number of model *k* is an integer ranging from 2 to 15, and α=1200.
3. First, the acoustic signal is decomposed using VMD for each group of *k* values. Second, the signal is decomposed into *k* BIMF components. Third, the mutual information between every BIMF component and the original signal is calculated. Finally, the mutual information is normalized.
4. According to the value of mutual information, the BIMF components are sequenced, and the screening coefficient is calculated according to the screening principle of BIMF components. The BIMF component larger than the screening factor is selected for signal reconstruction, and the BIMF component larger than the screening factor is the effective BIMF component. The mutual information screening coefficient is calculated as follows:
  (8) $$I\geq \frac{\max_{\mathrm{mutual}\;\mathrm{information}}}{10*\max_{\mathrm{mutual}\;\mathrm{information}}-3}$$
  where max_mutual information_ is the maximum mutual information value between all BIMF components. The reconstruction signal is reconstructed by summing the BIMF components with a mutual information value greater than *I*.
5. The root mean square error of the reconstructed signal and the original signal is calculated for different *k* values. The minimum root mean square error is determined, and *k* is used as the optimum result. The optimal value of the VMD decomposition parameter *k* is obtained based on the minimum root mean square error, and the effective BIMF components are obtained according to the mutual information and the mutual information screening coefficient. The SPL is used to transform the decomposed BIMF components.

### 4.4. Step 4: Acoustic Energy Flow Density

The acoustic energy flow density was extracted as eigenvalue vectors from the signal by the SPL.

### 4.5. Step 5: Classification and Recognition

The acoustic energy flux density eigenvectors were input to three different classifiers (SVM, KNN, and RF) to classify the siphon conditions.

## 5. Signal Processing

### 5.1. Wavelet Packet Transform

Seven siphon conditions were simulated in the hydraulics laboratory at the University of Bradford, including (1) a clean siphon with no damage; (2) a siphon with variable degrees of blockage; (3) a siphon with 4 different artificial leakages; and (4) a siphon with joint unsealed.

**Figure 5:**
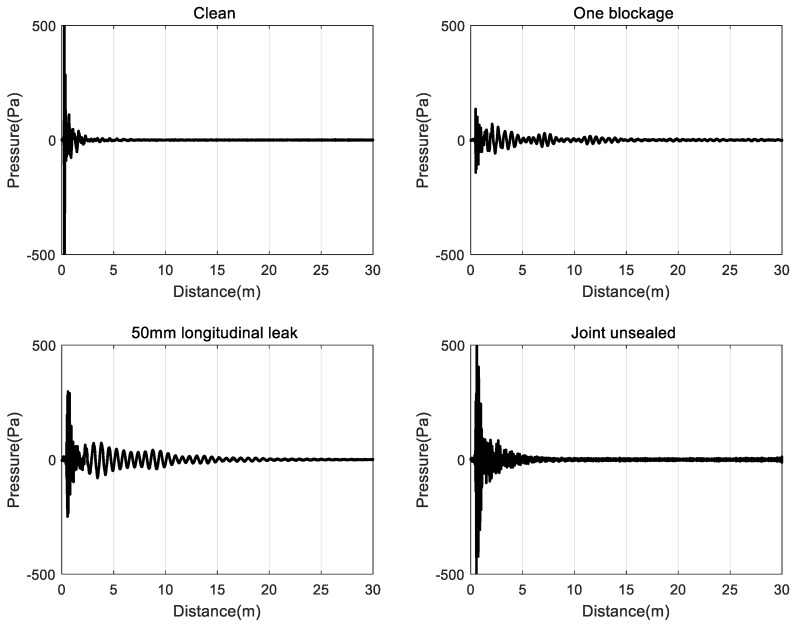
The acoustic pressure impulse response of four conditions in the siphon.

As can be seen from Figure 5, the data received by the hydrophone are remarkably similar and four conditions in the siphon can hardly be visually detected from the impulse response. An important component of data pre-processing is to modify the measured data to ensure that they were the more suitable for feature extraction and classification. Figure 5 shows that the characteristics of the acoustic signals in the four conditions in the siphon, including strong attenuation, non-periodicity, non-linearity, and non-stationarity. With the increase in the propagation distance of the acoustic waves in the siphon, the signal amplitude and the acoustic energy decreases. There is no obvious difference in time domain in the acoustic pressure impulse response, and it is impossible to distinguish blockage or leakage. Therefore, the sound pressure signal should be further processed.

In the laboratory experiment, the siphon was buried in sand and soil containing water. Therefore, the acoustic signals had a lot of noise. In the process of signal denoising, the noise in the signal was removed, and the original signal was restored.

**Figure 6:**
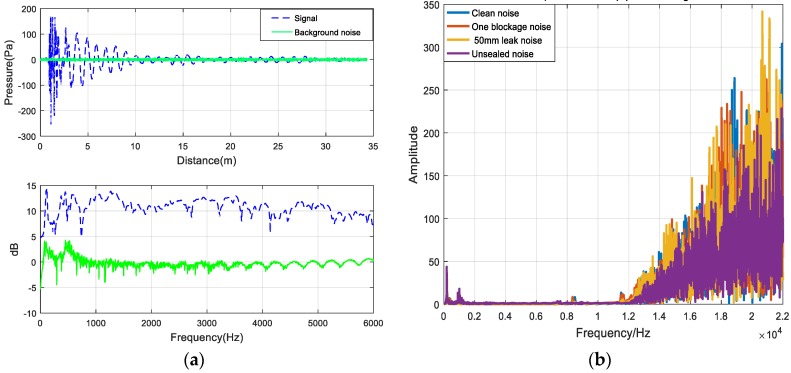
The noise of siphon conditions. (**a**) Environmental noise in the time domain and frequency domain. (**b**) The noise of four kinds of siphon conditions.

Figure 6a describes the time-domain and frequency-domain diagrams of the background noise and experimental acoustic signals in the frequency range of [0–4000] Hz. The frequency-domain diagram shows that the amplitude of the background noise is lower than that of the acoustic signals in the low-frequency band, indicating that the noise appears mainly in the high-frequency band. Figure 6b shows the frequency-domain diagram of the environmental noise in the four conditions. The frequency band of the noise signals is mainly concentrated in the range of [12000–22000] Hz. Therefore, four frequency bands decomposed by the wavelet packet are selected for signal reconstruction, namely, [0–2756], [2756–5512], [5512–8268], and [8268–11024]. The reconstructed signal is the desiccated signal, the purpose of which is to remove noise from the original signal.

### 5.2. Sound Pressure Level

**Figure 7:**
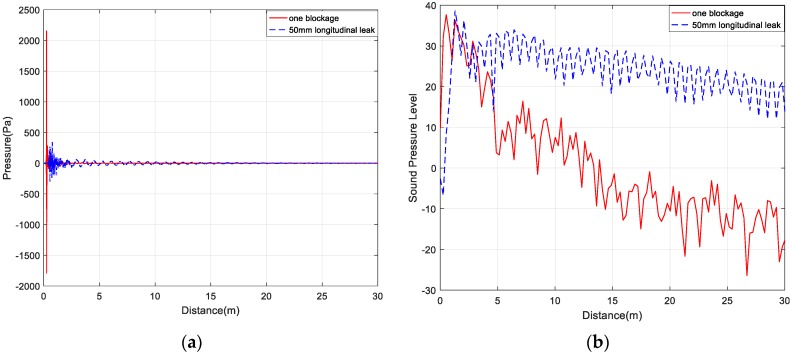
(**a**) Sound pressure of one blockage and 50 mm longitudinal leak; (**b**) SPL of one blockage and 50 mm longitudinal leak.

Due to the influences of transmission path and background interference, the signal has a low signal-to-noise ratio, and therefore it is difficult to accurately identify the sound pressure waveform shown in Figure 7a. By determining the SPL, the components with lower amplitude are emphasized to observe the characteristic signals hidden in the low-amplitude noise. Different conditions of the siphons can be discriminated, thereby facilitating feature extraction in the subsequent decomposition.

Figure 7a shows the sound pressure of the one blockage and the 50 mm longitudinal leak in the siphon. There is no obvious difference between the two conditions of the siphon, and it is difficult to distinguish the inflection point of the signal. As shown in Figure 7b, the inflection point of the signal can be clearly distinguished after the transformation into the SPL. The change of the waveform into the SPL shows that the sound pressure level of the blocking signal is higher than that of the siphon leak. WPT and VMD can remove the high-frequency noise in signals. The SPL can better reflect the characteristics of signals than the sound pressure signal and improves the ability to discriminate between different conditions.

### 5.3. VMD Decomposition

**Figure 8:**
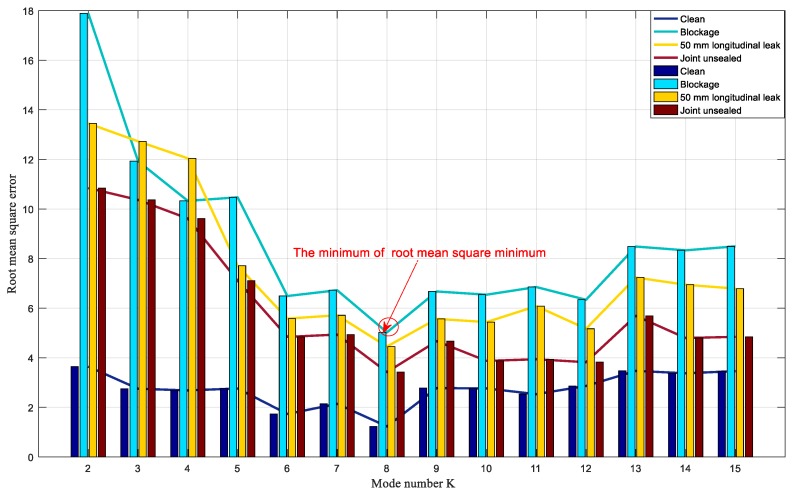
Root mean square error of four conditions in the siphon for different mode number K.

VMD was performed on the acoustic signal, and the mode number *k* of the decomposition ranged from two to 15. First, the mutual information between each BIMF component and the original signal was calculated and normalized in different *k* values. Second, mutual information screening coefficients were calculated, and the BIMF components larger than mutual information screening coefficients were selected for signal reconstruction. Finally, the mean square error of the reconstruction signal and the original signal was calculated. The minimum of the mean square error was the optimal mode number *k*. The VMD parameters were selected, and the sound pressure signals of the seven siphon conditions were decomposed using VMD. Similarly, the mutual information and the root mean square error of one sample in the four siphon conditions are calculated (clean siphon with no damage, siphon with different amounts of blockage, siphon with 55 mm longitudinal artificial leak, and joint unsealed). The results are shown in Figure 8.

As shown in Figure 8, as the *k* value increases from two to seven, the root mean square error is decreased. The decomposed acoustic signals belong to owe decomposition. If the preset scale *k* is small, the low-frequency band is classified as a high frequency band, and the effective BIMF components cannot be determined. When *k* = 8, the root mean square error value is minimum. The root means square error values in four siphon conditions, namely, clean siphon with no damage, siphon with different amounts of blockages, siphon with the 50 mm longitudinal leak, and siphon with the joint unsealed is 1.2303, 5.0244, 4.4517, and 3.4211 respectively. As the *k* value increases from eight to nine, the root means square error values of the four siphon conditions increase, and the acoustic signals are over-decomposed. Therefore, the most effective component for the four siphon conditions is *k* = 8.

**Figure 9:**
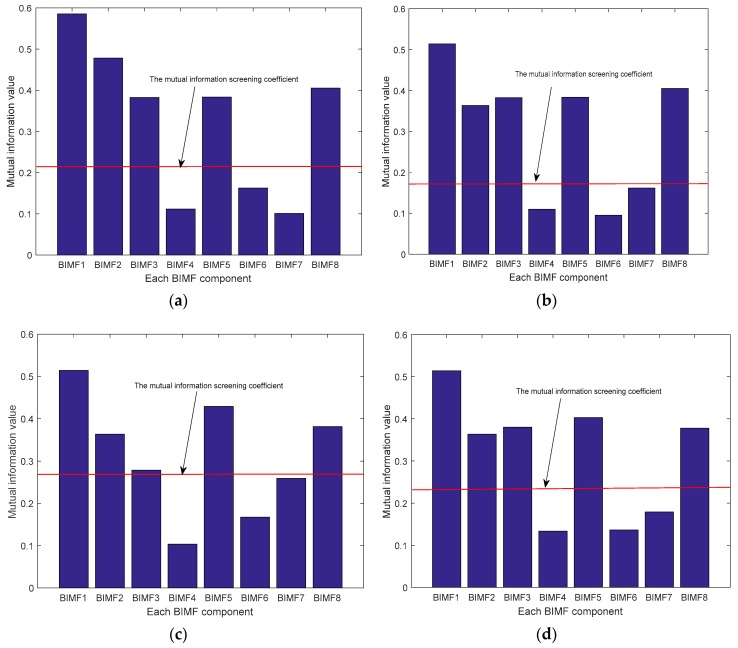
Mutual information of the four conditions in the siphon for 8 BIMF components. (**a**) clean; (**b**) blockage; (**c**) 55 mm longitudinal leak; (**d**) joint unsealed.

When K = 8, the mutual information and screening coefficients of the eight BIMF components of the four siphon conditions are shown in Figure 9. Mutual information screening coefficients of the four siphon conditions are 0.2052, 0.2732, 0.2733, and 0.2734, respectively.

As can be seen from Figure 9a, five BIMF components are larger than the screening coefficient, which are BIMF1, BIMF2, BIMF3, BIMF5, and BIMF8, respectively. These five components are effective components. Similarly, the five effective BIMF components of the other three conditions in the siphon are the same.

**Figure 10:**
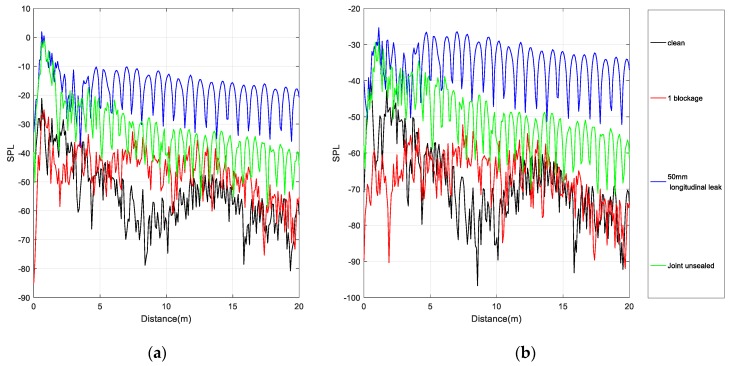
VMD components for four conditions of the siphon. (**a**) BIMF1; (**b**) BIMF2.

The acoustic signal of the siphon condition was denoised using WPT. WPT was used to decompose the acoustic signals into three-layer wavelet packets. By referring to the background noise signals for different siphon conditions, the appropriate wavelet packet frequency band was selected for signal reconstruction. Based on the mutual information and the root mean square error, the decomposition layers and the effective components of the VMD were selected. The BIMF components were transformed into SPL. The BIMF1 and BIMF2 components are shown in Figure 10.

The results in Figure 10 clearly show the differences of the SPL in the siphon conditions with/without damage. Particularly, the SPL generally show more differences between the conditions of 50 mm longitudinal leak and joint unsealed. The SPL obtained from the acoustic signals and filtered by VMD do not show a clear distinction in the conditions with clean siphon and one blockage in the siphon.

### 5.4. Acoustic Energy Flow Density

Acoustic waves reflect the mechanical vibration propagating in a medium. The waves are propagated in all elastic media, and the propagation characteristics are dependent on the nature of the medium. When sound waves propagate in a siphon, energy is transferred between the particles and the medium in an elastic or quasi-elastic collision effect. Therefore, the composition, shape, density, and motion state of the medium determine the changes in acoustic energy. If there is blockage or damage in the siphon, the propagation medium and the energy change. The acoustic energy flow density reflects different siphon conditions.

**Figure 11:**
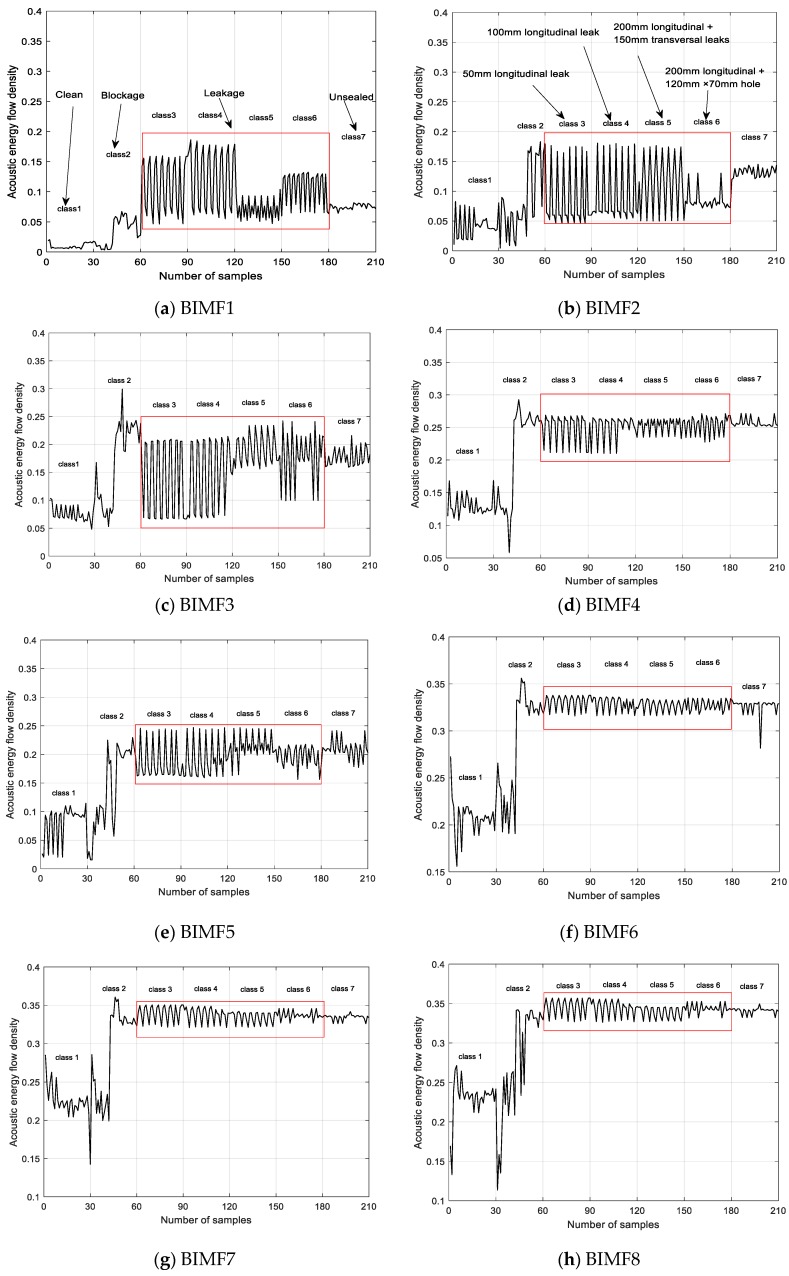
Eigenvectors of eight BIMF components in seven conditions of the siphon.

The characteristic vector set of the acoustic energy flow density of each BIMF component is calculated. There are seven siphon conditions, the sample number of each condition is 30, with a total of 210 samples. The sound energy flow density of eight BIMF components is shown in Figure 11. Herein, class 1–7 respectively represents: (1) clean; (2) siphon with a controlled amount of blockage; (3) a 50 mm longitudinal leak; (4) a 100 mm longitudinal leak; (5) 200-mm longitudinal and 150-mm transversal leaks; (6) a 200-mm longitudinal leak and a 120 mm × 70 mm hole; and (7) joint unsealed.

Figure 11 shows that the acoustic energy flux density of the 210 samples reflects different siphon conditions. If blockages occur in the siphon, the acoustic energy flux density exhibits a significant change. Figure 11 shows that the acoustic energy flux density tends to become more chaotic as the blocking degree increases, indicating that the acoustic energy flux density is sensitive to blockage. As the acoustic wave propagates in the siphon and encounters different types of leaks, a series of signals are reflected uniformly in the frequency range. When encountering blockages, the acoustic wave reflects the signal intensity in a non-uniform mode. 

Four conditions in the red wireframe represent different degrees of siphon leakage. BIMF1, BIMF2, BIMF3, BIMF5 and BIMF8 components change significantly in the red wire frame, which can represent the different leakage condition of siphons. The characteristics of some signals for different leakage degrees are similar because acoustic signals can be affected by type, shape, and size of leakage, flow rate, siphon pressure, surrounding environment, siphon material, pipe diameter, and water. It can be seen from Figure 11a–c,e,h that the acoustic energy flow density of the seven conditions differ obviously. At the same time, the effectiveness of the five BIMF components is verified.

## 6. Classification Results and Discussion

In order to verify the suitability of the VMD and acoustic energy flux density for identifying the siphon condition, three classification methods were presented, namely SVM, KNN, and RF. The experimental results listed in Table 1 were validated by 10-fold cross-validation of the average partition. The average accuracy of the 10-fold cross-validation was calculated. In the unfiltered VMD, all features representing the 8 BIMF components were input into the classifier, and the components BIMF1, BIMF2, BIMF3, BIMF5, and BIMF8 were filtered by the mutual information.

According to the experimental results, the model based on mutual information screening of the VMD components has a recognition accuracy of 96.5%, and the lowest accuracy is 94.4%. The unscreened model has the highest recognition accuracy of 87.8%. For the same classifier, the model results of the screened BIMF components based on mutual information are better than those of the unscreened BIMF components. The classification results show that the model which screens the BIMF component based on mutual information can accurately identify the siphon conditions. Therefore, our classification results indicate that the use of acoustic features and a suitable feature selection method result in better classification performance.

**Figure 12:**
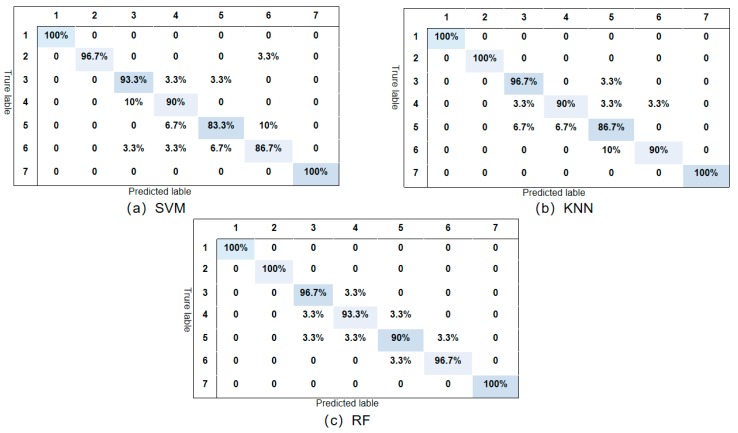
Average recognition accuracy of seven conditions in siphon.

As shown in Figure 12, in the conditions of clean siphon and joint unsealed, the classification accuracy and the recognition accuracy are 100%. The classification accuracy in the conditions of the 200 mm longitudinal, 150 mm transversal, and 200 mm longitudinal leaks and the 120 mm × 70 mm hole is the lowest. Due to the complexity of the siphon topology, the testing environment, the siphon environment, the blockage form, and the diversity of acoustic propagation characteristics, further research is required if multiple siphon leakages are present. Future research should consider the transmission characteristics of sound waves in different siphons and the attenuation behaviors of the acoustic wave propagation model using a blockage probability prediction model. Moreover, it is necessary to investigate data mining and to explore multiple closings of siphons and data pre-processing, data filtering, data selection, and other methods. In the acoustic data set in this study, important characteristics of the leakage degree and the conditions were extracted effectively and accurately.

## 7. Conclusions

In this study, a siphon was subjected to nondestructive testing using an acoustic approach to determine leakage, blockage, and joint unsealed conditions. Compared with other methods, the proposed active acoustic testing method has the advantages of quicker detection, simpler operation, longer detection distance, and lower cost. We used the VMD method combining mutual information and the root mean square error was used for evaluation. The method overcomes the common problems of mode aliasing and component leakage in traditional decomposition methods, and provides reliable signal component results.

1. Aiming to resolve the problem that some BIMF components after VMD decomposition contain the pipeline operation signal, a component screening method based on the mutual information screening coefficient is proposed to effectively extract the feature components containing a large amount of pipeline operation status information. The useful information in the signal is retained, which plays a crucial role in the in-depth mining of information.
2. The acoustic signal is transformed into SPL, and the SPL waveforms of the two signals are compared. It is found that SPL can effectively reflect the local characteristics of pipeline operation from mixed signals and increase the discrimination between different operating states.
3. We discussed the theoretical basis for acoustic signal processing with strong aliasing and low signal-to-noise ratio, and extracted the acoustic energy flux density as an eigenvector, which was input to different classifiers. The recognition accuracy of the proposed method is higher than 94.4%. The use of different classifiers indicates that the acoustic energy flux density is suitable for distinguishing different siphon conditions.

## Figures and Tables

**Table 1:** Average recognition accuracy after screening the VMD components based on mutual information.

Classifier	Unscreened Recognition Rate of BIMF Component (%)	Recognition Rate of BIMF Component Screened with VMD (%)
SVM	81.1%	94.4%
KNN	85.6%	94.7%
RF	87.8%	96.5%
